# Modeling and Experimental Study of Double-Row Bow-Type Micro-Displacement Amplifier for Direct-Drive Servo Valves

**DOI:** 10.3390/mi11030312

**Published:** 2020-03-16

**Authors:** Guoping Liu, Zhongbo He, Guo Bai, Jiawei Zheng, Jingtao Zhou, Ming Chang

**Affiliations:** 1 Army Engineering University Shijiazhuang Campus, Shijiazhuang 050003, China; luori810115@163.com; 2 Unit 63926 of PLA, Beijing 100192, China; bgbaiguobg@163.com; 3 Unit 32184 of PLA, Beijing 100072, China; 13227383720@163.com; 4 Department of Mechanized Infantry, Shijiazhuang Branch, Army Infantry Academy, Shijiazhuang 050083, China; 603254431@163.com

**Keywords:** micro-displacement amplifier, giant magnetostrictive actuator (GMA), pseudo-rigid model, servo valve

## Abstract

Giant magnetostrictive actuators (GMA) are widely used in the field of servo valves, but the displacement of GMA is limited, which renders meeting the requirements of large flow direct-drive electro-hydraulic servo valves (DDV) difficult. In order to solve these problems, this study proposes a double-row bow-type micro-displacement amplifier (DBMA), used to increase output displacement of GMA to meet the requirements. This study, by static analysis, analyzes the force of a flexure hinge based on theoretical mechanics and material mechanics, derives the stiffness matrix of the flexure hinge by the influence coefficient method, establishes the pseudo-rigid model, and derives the amplification ratio of a DBMA. Also, by kinetic analysis, using Castigliano’s second theorem, a formula of equivalent stiffness and natural frequency of DBMA were derived and the influences of different parameters on them were analyzed, respectively. After that, we analyzed the amplifier using finite element method (FEM) simulation software and verified the model by manufacturing a prototype and building a test system. Theoretical calculations and experimental results showed that the amplification ratio of the DBMA fluctuated between 15.43 and 16.25. The natural frequency was about 305 Hz to 314 Hz and the response bandwidth was up to 300 Hz. The error among the theoretical, simulated, and experimental values was within 8%, supporting the accuracy of the model.

## 1. Introduction

Giant magnetostrictive material (GMM) possesses excellent mechanical properties and frequency response characteristics [1,2]. Giant magnetostrictive actuators (GMA), which are designed with GMM as their core, have the faster response and larger output displacement than actuators made of similar materials (such as piezoelectric actuator and shape memory alloy actuator), etc. [3] With great load capacity, GMA exhibit broad application prospects in the field of electro-hydraulic servo systems. Based on GMA, the new Direct Drive Servo Valve (GMM-DDV) can improve response speed and reliability under the premise of ensuring a certain flow rate [4,5,6,7,8,9,10]. However, in respect to output requirements, the output displacement of GMA is limited, usually micrometers, and it is difficult to directly drive a mechanism that requires a large stroke. Therefore, an appropriate micro-displacement amplifier must be designed [11,12,13].

At present, micro-displacement amplifiers used in the field of precision machinery can be roughly divided into mechanical type and hydraulic type. Among them, the hydraulic amplifier is mainly based on Pascal’s theorem. Hwan-Sik Yoon et al. developed a hydraulic amplifier for piezoelectric actuators and verified it experimentally [14]. Yu et al. improved the hydraulic amplifier with a diaphragm structure and applied it to direct drive servo valves; the displacement amplification ratio was 9, and the governing error of valve spool was less than 1% [15]. Mechanical amplifiers usually feature a flexure hinge structure, which has the advantages of no back stroke, no friction, smooth motion, and high resolution. Paros and Weisbord derived a design formula for flexure hinges [16,17]. Sujit designed and manufactured a micro-displacement amplification mechanism based on the triangle principle, established a pseudo-rigid body model, and performed FEM simulations and experiments. The actual magnification was about 1.5 times [18]. Huang designed a diamond-shaped micro-displacement amplifier with bidirectional active output that features a symmetrical structure design and uses 4 actuators [19]. Huang et al. designed a skew-symmetric amplifier that converts a linear input into an angular output [20]. Lobontiu et al. used Castigliano’s second theorem to derive a closed-loop analytical formula of the stiffness of the bow-type amplifier and studied the influence of the relevant geometric parameters on its stiffness [21]. Wan used the kinetic energy theorem to consider the stiffness of the overall moving pair and the rotational stiffness of the flexible hinge, and derived the parameters affecting amplifier displacement and amplification ratios [22]. Muraoka used a honeycomb design and proposed an array-type mechanical amplifier with a more compact structure [23]. Wei used a hybrid flexible hinge to design a bow-and-pin magnification mechanism with multi-stage magnification [24]. Ling et al. designed a two-stage bow-type amplifier with a large range and high frequency and established a displacement amplification ratio formula by defining the impedance factor [25,26]. Zhang et al. designed a bow-type amplifier and established its simplified model [27]. Ye et al. used the matrix method to establish the flexibility matrix of the bow-type amplifier and derived its output displacement formula and amplification formula [28,29]. The theory of micro-displacement amplifiers for valves has developed from simple studies of stress-strain relationships to the comprehensive utilization of the law of energy, Castigliano’s second theorem, vibration theory, and matrix theory, which have much improved theoretical research and made models more precise and accurate. Nevertheless, the amplifier requires too much space, as compared with the actuator, and the space utilization rate is too low [30]. Therefore, the structural design needs to be optimized to improve the space utilization rate.

## 2. Static Analysis of Double-Row Bow-type Micro-Displacement Amplifier (DBMA)

According to the principle of energy conservation, when the output energy *W* of the GMA is determined, the amplification ratio *R* provided by the micro-displacement amplifier is larger, and the force *F_out_* at the output end of the amplifier is smaller, as in Equation (1):

(1) $$\left\{ \begin{array}{l} W=x_{in}\times F_{in}=x_{out}\times F_{out}+W_{l}\\ x_{out}=x_{in}\times R\\ F_{out}\leq F_{in}/R \end{array}\right.$$

where *x_in_* and *F_in_* are respectively the displacement and force provided by the GMA, *x_out_* is the output displacement of the amplifier, and *W_l_* is the loss during amplification. Therefore, the amplification ratio of the micro-displacement amplifier cannot be unrestricted, and the optimum amplification ratio should be reasonably selected in consideration of practical applications.

**Figure 1:**
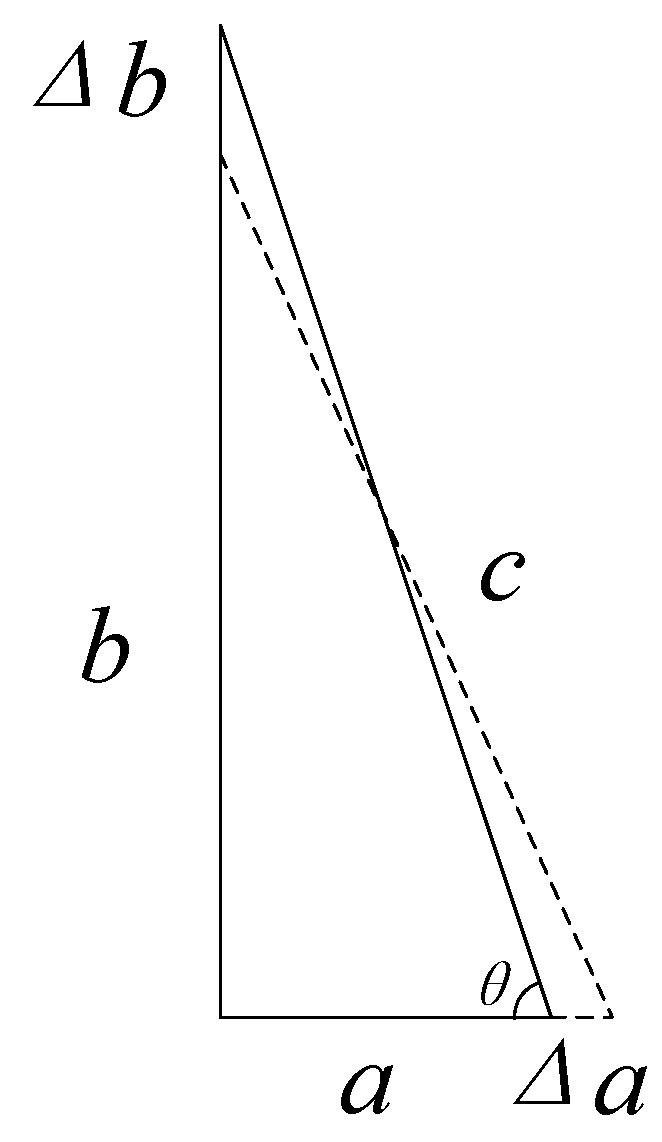
Amplification method based on the Pythagorean theorem.

The basic principle of the bow-type micro-displacement amplifier is the Pythagorean theorem, as shown in Figure 1.

The three sides of the right triangle are *a*, *b,* and *c*, and the length of the hypotenuse c is constant. When the leg *a* is elongated by *Δa*, the leg *b* is shortened by *Δb*. *θ* is the angle between the shortest leg and the hypotenuse in the triangle.

According to Pythagorean theorem

(2) $$\left\{ \begin{array}{l} a^{2}+b^{2}=c^{2}\\ {\left(a-\Delta a\right)}^{2}+{\left(b+\Delta b\right)}^{2}=c^{2} \end{array}\right.$$

Ignoring the second order infinitesimal, the amplification rate is

(3) $$\lambda =\frac{\Delta a}{\Delta b}\approx \frac{b}{a}=\tan\theta ,$$

When *b* > *a*, *λ* > 1. Therefore, in the amplifier design process, when the hypotenuse is unchanged, the long leg should be elongated, and the short leg should be shortened.

**Figure 2:**
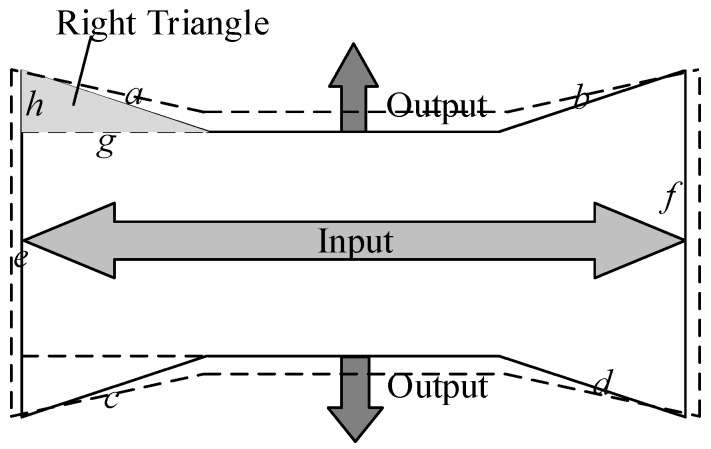
Simplified diagram of a bow-type amplifier.

The bow-type amplifier is shown in Figure 2. Four oblique sides *a*, *b*, *c*, *d*, and hypotenuses *e* and *f* form four right triangles. The length of the hypotenuse *a* is constant; when the input end elongates, the leg *g* of the triangle elongates as well, and the leg *h* shortens as a result. This means that the output end outputs displacement to both sides, and the ratio of displacement amplification depends on the angle between the sides *a* and *h*. 

**Figure 3:**
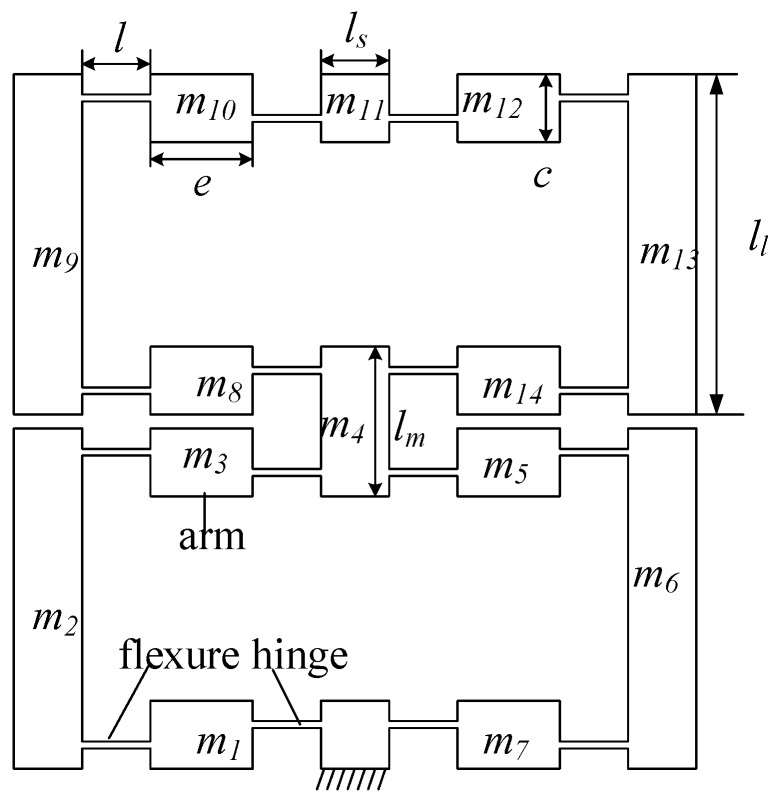
Structure of the double-row bow-type micro-displacement amplifier (DBMA).

DBMA is shown in Figure 3. In order to increase the amplifier’s amplification ratio and meet the output force requirements simultaneously, the two bow-type amplifiers are used in series.

**Figure 4:**
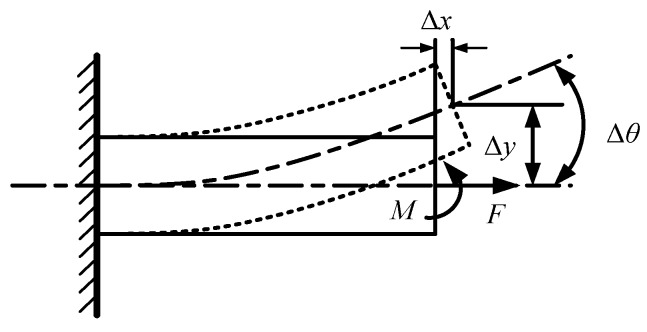
The working principle of the flexure hinge.

In DBMA, a large dimensional difference exists between the flexure hinge and the arm, and the deformation concentrates on the flexure hinge. Therefore, the arm can be regarded as a rigid body, and the flexure hinge can be regarded as a flexible rod, or a thin plate fixed at both ends. The working principle of the flexure hinge is shown in Figure 4.

It shows that, when the flexure hinge is subjected to the horizontal force *F*, a horizontal deformation *Δx* will occur; when subjected to the moment *M*, a vertical deformation *Δy* and an angle *Δθ* will occur.

**Figure 5:**
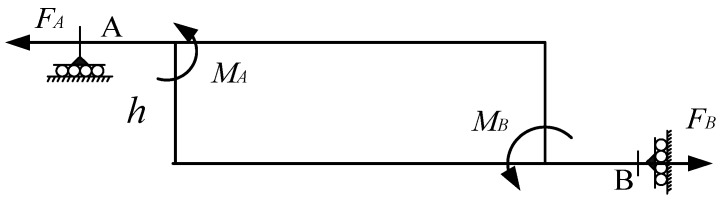
The force of the arm.

The force of the arm is shown in Figure 5.

The two sides of the arm are respectively subjected to the horizontal forces *F_A_* and *F_B_*, which generate the moments *M_A_* and *M_B_* at the flexure hinges A and B. It is easy to draw conclusions from the static balance theory:

(4) $$\left\{ \begin{array}{l} F_{A}=F_{B}\\ 2M_{A}=2M_{B}=F_{A}\cdot h \end{array}\right.$$

**Figure 6:**
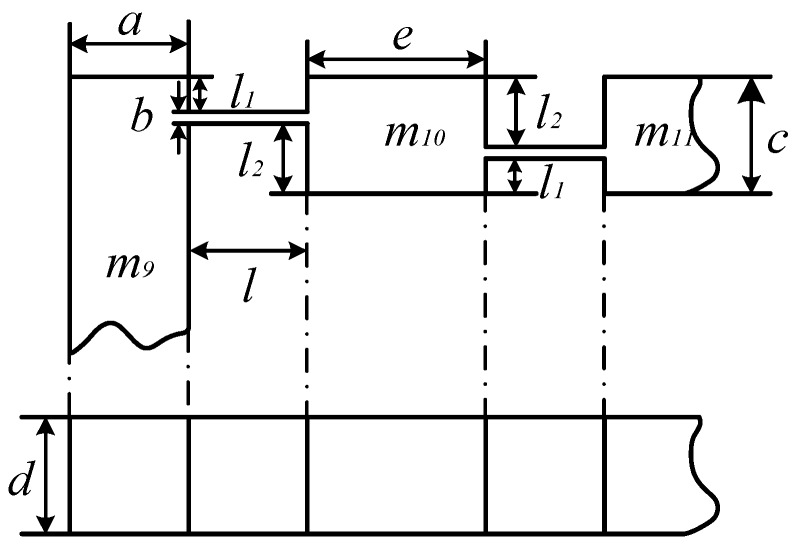
DBMA’s 1/8 model.

Since DBMA is a symmetric structure, its 1/8 model is chosen, as shown in Figure 6.

Since the output displacement of the GMA is small compared to the size of the amplifier, the bending angle *Δθ* of the flexure hinge is also small, so the chord length causing by the rotation of the arm is approximately equivalent to the arc length. Therefore, it can be seen that under the action of the input force, the horizontal displacement *d_x_* and vertical displacement *d_y_* in 1/8 DBMA model are:

(5) $$\left\{ \begin{array}{l} d_{x}=2\Delta y+h\cdot \Delta \theta \\ d_{y}=2\Delta x+e\cdot \Delta \theta \end{array}\right.$$

Where, $\Delta x=\frac{F}{K_{lx}}$, $\Delta y=\frac{F}{K_{ly}}$ and $\Delta \theta =\frac{M}{K_{\theta }}$ are respectively the x-axis and y-axis deformation and the rotation deformation of flexure hinge, $h=l_{1}-l_{2}$ is the height difference between the two flexure hinges, $K_{lx}$ and $K_{ly}$ are respectively the stiffness of flexure hinge on the *x*-axis and *y*-axis, and $K_{\theta }$ is the angular stiffness of the supporting arm.

**Figure 7:**
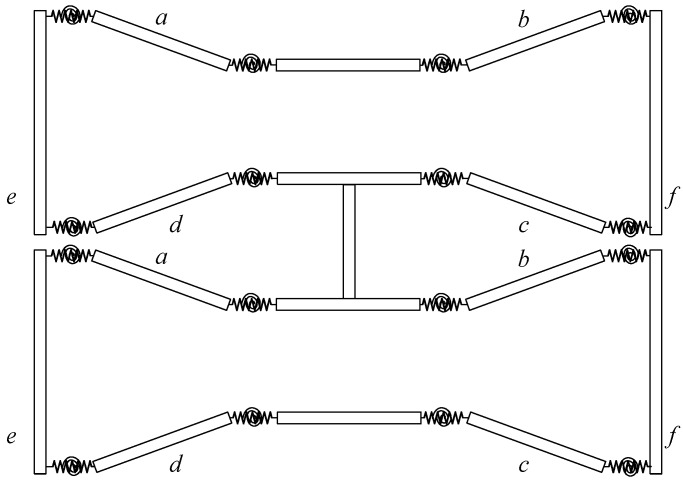
Pseudo-rigid body model of DBMA.

According to the pseudo-rigid-body model, the deformation of the flexure hinge can be regarded as a stretch-bend combination deformation, which can simplify the flexure hinge as a combination of a tension spring and a torsion spring. The pseudo-rigid-body model of DBMA is shown in Figure 7.

**Figure 8:**
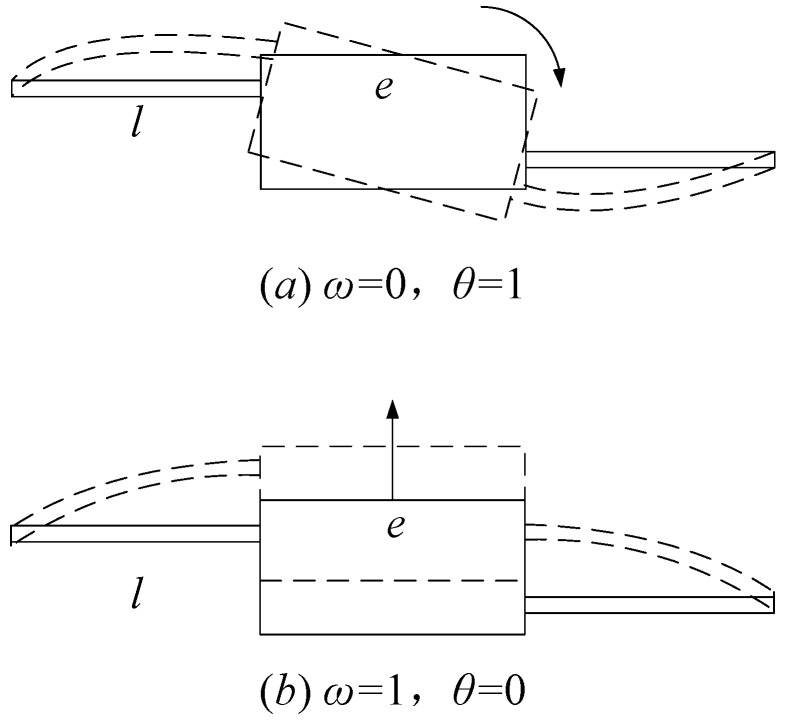
The influence coefficient method. (**a**) *ω* = 0, *θ* = 1 (**b**) *ω* = 1, *θ* = 0.

Using the influence coefficient method, as shown in Figure 8, the stiffness coefficient matrix of the hinges at both ends of the arm can be obtained.

In Figure 8a, *ω* = 0, *θ* = 1; in Figure 8b, *ω* = 1, *θ* = 0. So the stiffness coefficient matrix is

(6) $$K=\left(\begin{array}{cc} \frac{12EI_{z}}{l^{3}} & 0\\ 0 & \frac{2(2l+e)EI_{z}}{l^{2}} \end{array}\right)$$

where *EI_z_* is the bending stiffness of the flexure hinge, *l* and *e* are the length of the flexure hinge and the arm respectively, *E* is the elastic modulus of the material, $I_{z}=\frac{db^{3}}{12}$ is the moment of inertia of the flexure hinge, and *b* is the thickness of the flexure hinge, *d* is the thickness of the amplifier (also the width of the flexure hinge).

Therefore, the amplification ratio of DBMA is

(7) $$R=\frac{4d_{y}}{d_{x}}=\frac{4l(3h^{2}+8l^{2}+4el)}{4b^{2}e+8b^{2}l+3ehl}$$

The partial derivatives of *l*, *h*, *e*, *b* to *R*, respectively, is

(8) $$\left\{ \begin{array}{l} \frac{\partial R}{\partial l}=\frac{16(8b^{2}e^{2}l+3b^{2}eh^{2}+32b^{2}el^{2}+32b^{2}l^{3}+3e^{2}hl^{2}+12ehl^{3})}{{\left(4b^{2}e+8b^{2}l+3ehl\right)}^{2}}>0\\ \frac{\partial R}{\partial h}=\frac{12l(8b^{2}eh+16b^{2}hl-4e^{2}l^{2}+3eh^{2}l-8el^{3})}{{\left(4b^{2}e+8b^{2}l+3ehl\right)}^{2}}<0\\ \frac{\partial R}{\partial e}=-\frac{12hl(4b^{2}h+3h^{2}l+8l^{3})}{{\left(4b^{2}e+8b^{2}l+3ehl\right)}^{2}}<0\\ \frac{\partial R}{\partial b}=-\frac{32bl(e+2l)(3h^{2}+8l^{2}+4el)}{{\left(4b^{2}e+8b^{2}l+3ehl\right)}^{2}}<0 \end{array}\right.$$

**Figure 9:**
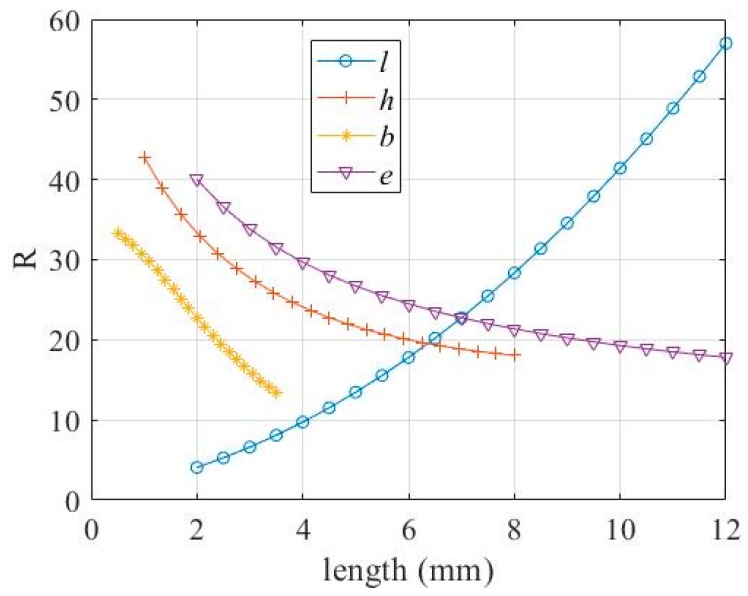
Relationship between *R* and *h*, *l*, *e*, *b.*

It can be seen from Equation (8) that in the positive range, *R* increases as *l* increases, and decreases as *h*, *e* and *b* increase. The relationship between *R* and the parameters *h*, *l, e* and *b*, respectively, is shown in Figure 9.

## 3. Kinetic Analysis of DBMA

Since the DBMA is used to drive the servo valve spool to move, it is necessary to pay attention to the output force change while considering the amplification ratio. Known as

(9) F=kl

When the output displacement *l* is a constant, the larger the stiffness *k*, the larger the output force *F*. In order to meet the demand for the output force of the DBMA, the equivalent stiffness *k* should be increased as much as possible under the demand of the output displacement.

The DBMA could be regarded as a mass-spring system, and its vibration equation can be expressed as

(10) $$M\ddot{u}+Ku=0$$

Consider the DBMA as a single-degree-of-freedom system, using its simplified model, as shown in Figure 6. According to the vibration theory, its natural frequency is

(11) $$f_{n}=\frac{1}{2\pi }\sqrt{\frac{K_{e}}{m_{e}}}$$

where *K_e_* and *m_e_* are respectively the equivalent stiffness and the equivalent mass of the DBMA.

According to the pseudo-rigid body theory, the flexure hinge can be regarded as an ideal rotating joint. As shown in Figure 7, the overall elastic potential energy of the system is

(12) $$U=16\left(\frac{1}{2}K_{lx}{\left(\Delta x\right)}^{2}+\frac{1}{2}K_{ly}{\left(\Delta y\right)}^{2}+\frac{1}{2}K_{\theta }{\left(\Delta \theta \right)}^{2}\right)$$

According to the Castigliano’s second theorem

(13) $$K\left(\begin{array}{c} u\\ \theta \end{array}\right)=\left(\begin{array}{c} F\\ M \end{array}\right)$$

where $u=\frac{\partial U}{\partial F}$ is the displacement of the arm in the *x*-axis direction, and *θ* is the arm rotation angle.

According to Equation (13), the flexure hinge coupling stiffness is

(14) $$K_{e}=\frac{Eb^{3}d(e+2l)}{32b^{2}l^{2}+16eb^{2}l+6h^{2}l^{2}+32l^{4}+16el^{3}}$$

The partial derivatives of $K_{e}$ with respect to *l*, *h*, *e*, *b*, *d*, respectively, are

(15) $$\left\{ \begin{array}{l} \frac{\partial K_{e}}{\partial l}=-\frac{Eb^{3}d(4b^{2}e^{2}+16b^{2}el+16b^{2}l^{2}+12e^{2}l^{2}+3eh^{2}l+48el^{3}+3h^{2}l^{2}+48l^{4})}{{l^{2}(16b^{2}l+8eb^{2}+3h^{2}l+16l^{3}+8el^{2})}^{2}}<0\\ \frac{\partial K_{e}}{\partial h}=-\frac{3Eb^{3}dh(e+2l)}{{\left(16b^{2}l+8eb^{2}+3h^{2}l+16l^{3}+8el^{2}\right)}^{2}}<0\\ \frac{\partial K_{e}}{\partial e}=\frac{3Eb^{3}dh^{2}}{2{\left(16b^{2}l+8eb^{2}+3h^{2}l+16l^{3}+8el^{2}\right)}^{2}}>0\\ \frac{\partial K_{e}}{\partial b}=\frac{Eb^{2}d(e+2l)(16b^{2}l+8eb^{2}+9h^{2}l+48l^{3}+24el^{2})}{2l{\left(16b^{2}l+8eb^{2}+3h^{2}l+16l^{3}+8el^{2}\right)}^{2}}>0\\ \frac{\partial K_{e}}{\partial d}=\frac{Eb^{3}(e+2l)}{32b^{2}l^{2}+16eb^{2}l+6h^{2}l^{2}+32l^{4}+16el^{3}}>0 \end{array}\right.$$

**Figure 10:**
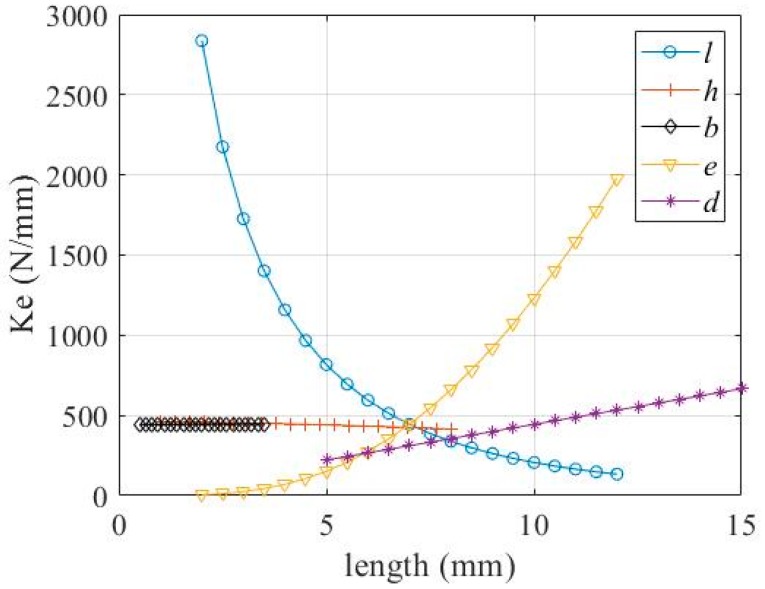
Relationship between *K_e_* and *h*, *l*, *e*, *b*, *d.*

It can be seen from Equation (15) that, in the positive range, *K_e_* decreases as *l* and *h* increase, and increases as *e*, *b*, and *d* increase. The relationship between *K_e_* and the parameters *h*, *l, e* and *b*, respectively, is shown in Figure 10.

The kinetic energy of the DBMA consists of the vibration along the *x*-axis and *y*-axis and the rotational kinetic energy around the z-axis of each arm.

(16) $$T=\frac{1}{2}\sum_{k=1}^{14}m_{k}\left({\dot{u}}_{x_{k}}^{2}+{\dot{u}}_{y_{k}}^{2}\right)+\frac{1}{2}\left(\sum_{k=1}^{14}J_{k}\omega_{k}^{2}\right)$$

where $m_{k}$ is the mass of each arm (where $m_{1}=m_{3}=m_{5}=m_{7}=m_{8}=m_{10}=m_{12}=m_{14}$, $m_{2}=m_{6}=m_{9}=m_{13}$), $u_{x_{k}}=2d_{x_{k}}$ is the displacement of each arm in the horizontal direction, and $u_{y_{k}}=2d_{y_{k}}$ is the displacement of each arm in the vertical direction.

The moment of inertia $J_{k}(k=1,3,5,7,8,10,12,14)$ and the angular velocity of rotation *ω* of the arm can be expressed as

(17) $$\{\begin{array}{l} J_{k}=\frac{1}{12}m_{k}\left[{\left(l_{2}-l_{1}\right)}^{2}+{\left(l+e\right)}^{2}\right]\\ \omega =\frac{{\dot{u}}_{y}}{4\sqrt{{\left(l_{2}-l_{1}\right)}^{2}+{\left(l+e\right)}^{2}}} \end{array}$$

Substituting Equation (17) into Equation (16), the following equation can be obtained.

(18) $$T=\left[\left(\frac{1}{R^{2}}+\frac{9}{4}\right)m_{1}+\left(\frac{2}{R^{2}}+\frac{5}{4}\right)m_{2}+\frac{m_{4}}{2}+2m_{11}\right]{\dot{u}}_{y}^{2}$$

Therefore, the equivalent mass of the DBMA is

(19) $$m_{e}=\left(\frac{1}{R^{2}}+\frac{9}{4}\right)m_{1}+\left(\frac{2}{R^{2}}+\frac{5}{4}\right)m_{2}+\frac{m_{4}}{2}+2m_{11}$$

According to Equations (14) and (19), the natural frequency of the DBMA is

(20) $$f_{n}=\frac{1}{2\pi }\sqrt{\frac{K_{e}}{m_{e}}}=\frac{1}{2\pi }\sqrt{\frac{\frac{Eb^{3}d(e+2l)}{32b^{2}l^{2}+16eb^{2}l+6h^{2}l^{2}+32l^{4}+16el^{3}}}{(\frac{1}{R^{2}}+\frac{9}{4})m_{1}+(\frac{2}{R^{2}}+\frac{5}{4})m_{2}+\frac{m_{4}}{2}+2m_{11}}}$$

The partial derivatives of $K_{e}$ and *R* to $f_{n}$, respectively, is

(21) $$\left\{ \begin{array}{l} \frac{\partial f_{n}}{R}=\frac{(m_{1}+2m_{2})\sqrt{Edb^{3}(e+2l)}}{2\pi R^{3}\sqrt{(32b^{2}l^{2}+16eb^{2}l+6h^{2}l^{2}+32l^{4}+16el^{3})m_{e}^{3}}}>0\\ \frac{\partial f_{n}}{K_{e}}=\sqrt{\frac{4b^{2}e+8b^{2}l+3ehl}{K_{e}l(3h^{2}+8l^{2}+4el)}}>0 \end{array}\right.$$

It can be seen from Equation (21) that the natural frequency *f_n_* of the DBMA is positively correlated with the amplification ratio *R* and the equivalent stiffness *K_e_*, that is, increasing either *R* or *K_e_*, the natural frequency can be increased.

## 4. Simulation Research on DBMA

The DBMA was analyzed by finite element method (FEM) software. The values of various parameters are shown in Table 1.

**Figure 11:**
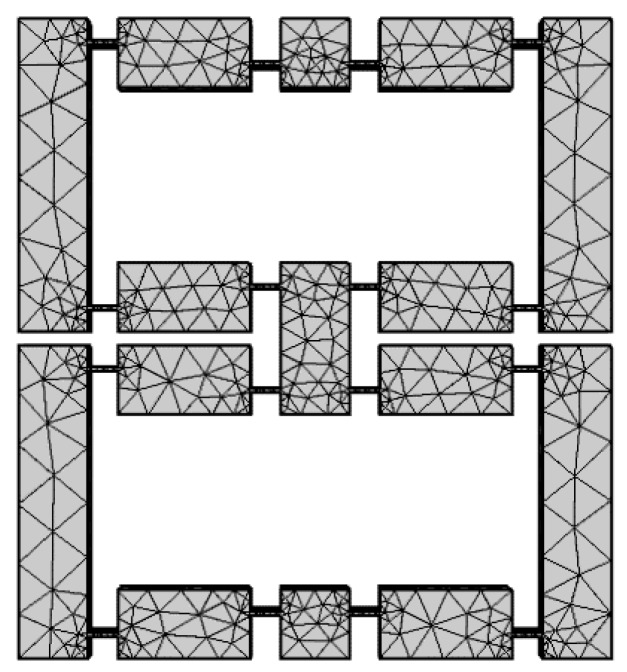
Meshing result in finite element method (FEM) software.

**Figure 12:**
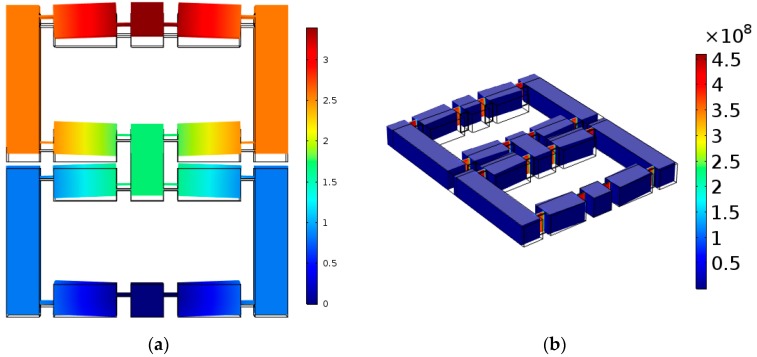
Static deformation simulation results in FEM software. (**a**) The deformation of DBMA, (**b**) The stress distribution of DBMA.

The meshing result is shown in Figure 11. And static deformation simulation results are shown in Figure 12.

**Figure 13:**
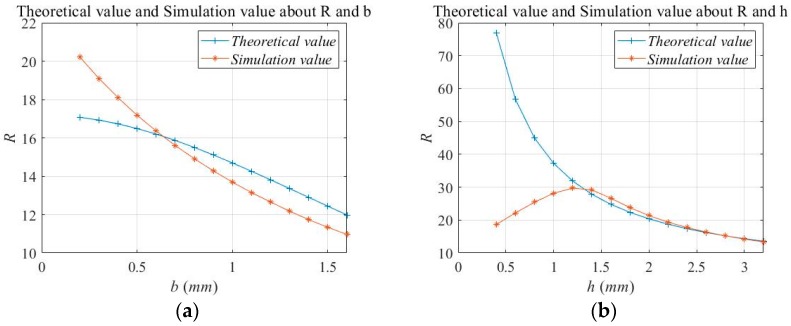
The effect of *b* and *h* on *R* respectively. (**a**) Theoretical and simulation values of *R* and *b*, (**b**) Theoretical and simulation values of *R* and *h*.

We selected different *b* and *h* for 30 FEM simulations; the theoretical values and simulation results were compared, as shown in Figure 13.

It can be seen from Figure 13 that when the thickness *b* of the flexure hinge changed, the theoretical value of the amplification ratio *R* fit the simulated value better, and the error was within 7%. With respect to the relationship between *h* and *R*, when *h* > 1.2 mm, the theoretical results were very close to the simulation results. When *h* < 1.2 mm, the theoretical value of *R* differed greatly from the simulated value. The simulated value reached the maximum point when *h* = 1.2 mm. In the theoretical calculation, *R* was negatively correlated with *h*. This was because when the *h* was small, the height difference between the two flexure hinges was also small, and the deformation mainly manifested as the tensile deformation of the flexure hinge. The properties of the material were taken into account in the simulation calculation, but were not included in the theoretical calculation, thus causing an error.

**Figure 14:**
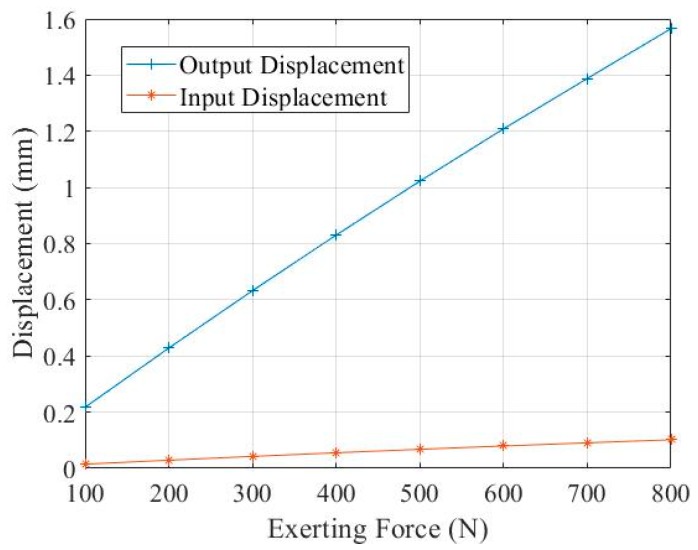
Relationship between input and output.

We selected *h* = 2.6 mm, *b* = 0.6 mm, re-established the model, applied a uniform force of 0–800 N, and recorded the input displacement and output displacement respectively, as shown in Figure 14. It can be seen from the analysis that the input displacement change of the DBMA was approximately linear with the output displacement change. Therefore, under the uniform force of 0–800N, the static deformation of the DBMA remained nearly unchanged, and the amplification ratio was stable at around 15.3. Compared with the theoretical calculation, the relative error was 6.06%.

**Figure 15:**
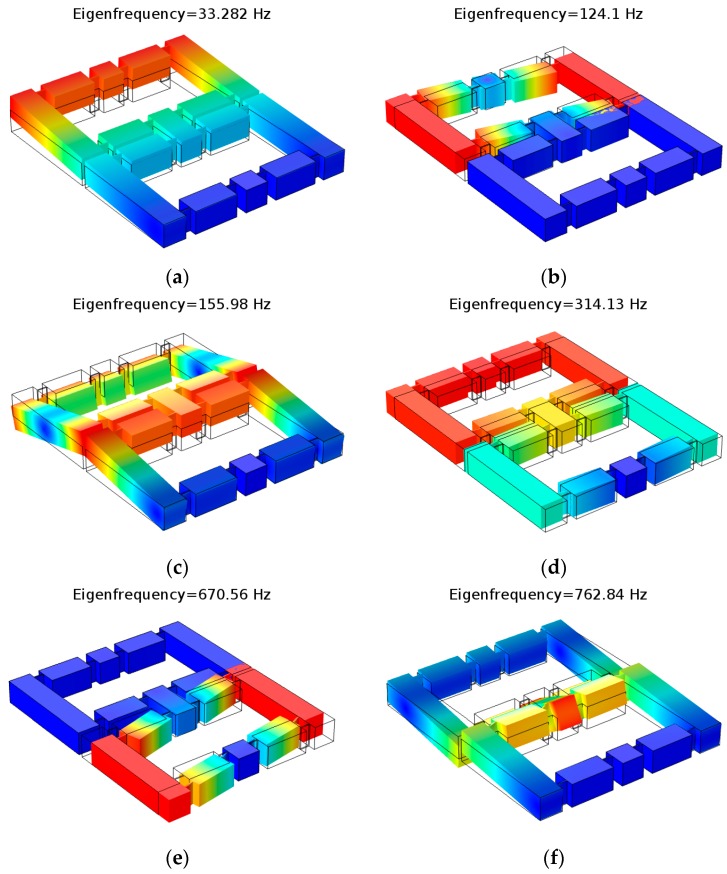
First six mode shapes. (**a**) first order mode shape, (**b**) second order mode shape, (**c**) third order mode shape, (**d**) fourth order mode shape, (**e**) fifth order mode shape, (**f**) sixth order mode shape.

Using the modal simulation function in the FEM software, the first six modes of the DBMA were obtained as shown in Figure 15.

It can be seen from Figure 15 that the fourth-order mode shape of the DBMA was the same as that of the single-degree-of-freedom model, and other mode shapes caused displacement in other directions. In the FEM simulation results, the frequency of the fourth mode was 314.13 Hz, and the natural frequency is 293.9 Hz in the theoretical calculation. The difference between them was 6.44%. The theoretical calculation was in good agreement with the simulation results.

## 5. Experimental Verification of DBMA

**Figure 16:**
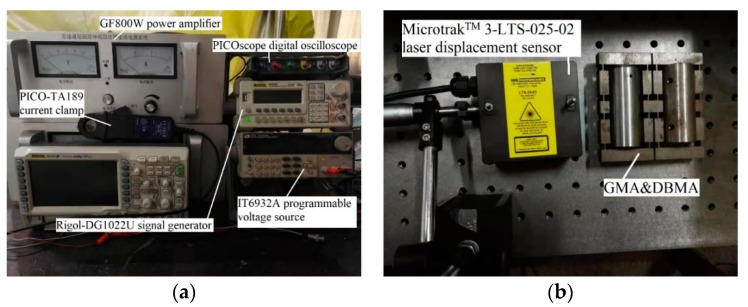
Test System. (**a**) Monitoring part of the test system, (**b**) Measuring part of the test system.

According to the theoretical calculation and simulation research, a DBMA-GMA prototype was made and a corresponding test system was built, as shown in Figure 16b.

As is shown in Figure 16, a Rigol-DG1022U signal generator (Shenzhen Keruijie Technology Co., Ltd., Shenzhen, China) was used to generate the excitation signal, which was amplified by the GF800W power amplifier (Army Engineering University, Shijiazhuang, China) to drive the DBMA-GMA. Displacement was measured using the Microtrak^TM^ 3-LTS-025-02 laser displacement sensor (MTI Instruments, Inc., Washington, USA), which was supplied by the IT6932A programmable voltage source. The input current value was monitored by the PICO-TA189 current clamp and the test data were acquired by the PICO scope digital oscilloscope (PicoTechnology Inc, Cambridgeshire, UK).

**Figure 17:**
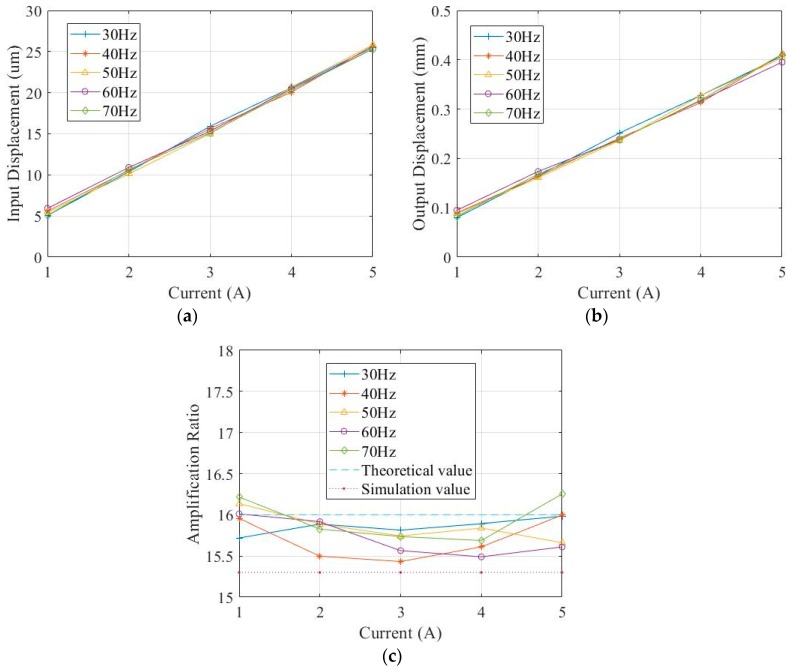
Response of sinusoidal excitation. (**a**) Input displacement & current, (**b**) Output displacement & current, (**c**) Amplification ratio & current.

A sinusoidal signal was used to excite the DBMA-GMA, with the signal frequency changing between 30 Hz and 70 Hz with 10 Hz as the gradient and the input current changed between 1 A–5 A with 1 A as the gradient, and the axial and transverse displacement of the DBMA-GMA is measured by the laser displacement sensor. During the experiment, 10 measurements were made for each point. The maximum and minimum value in the data were removed during processing, and the mean value of the remaining 8 data was taken. Finally, the displacement response curve of the DBMA-GMA under the excitation of the sinusoidal signal could be obtained as Figure 17 shows.

As can be seen from Figure 17, both the input and output displacements were approximately proportional to the current, with good linearity. It shows that in the range of 30–70 Hz, the dynamic performance of the DBMA was relatively stable, and the amplification ratio fluctuates between 15.43–16.25. The error between the experimental results and the theoretical calculation was between 1.6% and 3.56% and was between 2.81% and 8.17% from the simulation calculation.

**Figure 18:**
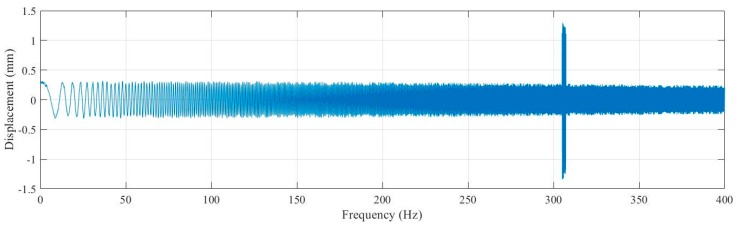
Displacement & Frequency.

Sinusoidal frequency sweep signal of 0–400 Hz is applied to the DBMA-GMA, and the current was set as 4A. After the measured data were sorted out, the relationship between frequency and displacement was obtained as shown in Figure 18.

It can be seen from Figure 18 that the measured natural frequency of the DBMA was 305.01 Hz, with an error of 3.67% from the theoretical calculation and 2.87% from the simulation result.

## 6. Discussion

In this study, we designed a DBMA and performed mathematical modeling, simulation modeling, and experimental verification. In the experimental verification, we chose a larger size prototype for analysis. There were two reasons for this choice: One was to avoid the influence of machining errors on the experimental accuracy; the other was that such a large amplifier was required due to the size of the giant magnetostrictive actuator. However, we believe that the size of the prototype had no effect on the accuracy of the model for the following reasons:

(a) In mathematical modeling, we did not discuss the size of the model, so mathematical models were established at the micron and millimeter levels. However, it should be noted that the condition for the assumption in Figure 4 was that the thickness of the arm needed to be much larger than the flexure hinge, in order to treat the arm as a rigid body, and the flexure hinge as a thin plate with fixed ends to simplify the model.
(b) In the simulation analysis, we actually established more than one size model. The simulation results show that when all the size parameters of the model were enlarged or reduced in proportion, there was no effect on the amplification ratio. When only a few parameters were changed, the results changed. These results are shown in Figure 9, Figure 10 and Figure 13.
(c) Since the size of the actuator of the giant magnetostrictive servo valve reached the millimeter level and an amplifier was used with the actuator, we did not make a smaller prototype for experiments. However, from the results of mathematical modeling and simulation, we believe that such structures and modeling methods would be correct at larger or smaller sizes.

## 7. Conclusions

- This paper establishes a pseudo-rigid model of DBMA, analyzes the stress form of flexure hinge, and derives the stiffness matrix of flexure hinge by the influence coefficient method.
- A static and kinetic analysis of DBMA was carried out, and a formula of displacement magnification was derived. Using Castigliano’s second theorem, a formula of equivalent stiffness and natural frequency of DBMA were derived. The effects of different parameters on the magnification, equivalent stiffness, and natural frequency were analyzed, respectively.
- A simulation of DBMA was carried out using FEM simulation software. A prototype of DBMA was fabricated, a corresponding test system was built, and experimental research was conducted. Errors of theoretical calculation, simulation results, and experimental data were all within 8%, which supported the correctness of the model.
- Theoretical research and experimental results show that the displacement amplification ratio of DBMA was stable at about 15.5, the natural frequency is about 305Hz to 314Hz, and the response bandwidth was up to 300 Hz, which satisfy the servo valve output requirements.

## Figures and Tables

**Table 1:** Size of DBMA for simulation.

Parameters	Description	Value
*c*	Width of arms 1,3,5,7,8,10,12,14	10 mm
*a*	Width of arms 2,6,9,13	10 mm
*d*	Thickness of the amplifier (also the width of the flexure hinge)	10 mm
*l*	Length of the flexure hinge	4.41 mm
*e*	Length of the arms 2,6,9,11	19.90 mm
*l_s_*	Width of arm 11	9 mm
*l_l_*	Length of arms 2,6,9,13	45.47 mm
*E*	Elastic modulus of the material	215 Mpa
